# Supplementary figures and images for: Short senolytic or senostatic interventions rescue progression of radiation-induced frailty and premature ageing in mice
Source: eLife. 2022 May 4;11:e75492. doi: 10.7554/eLife.75492 (PMC9154747; doi:10.7554/eLife.75492)

Y IR Met

NOX4 ->

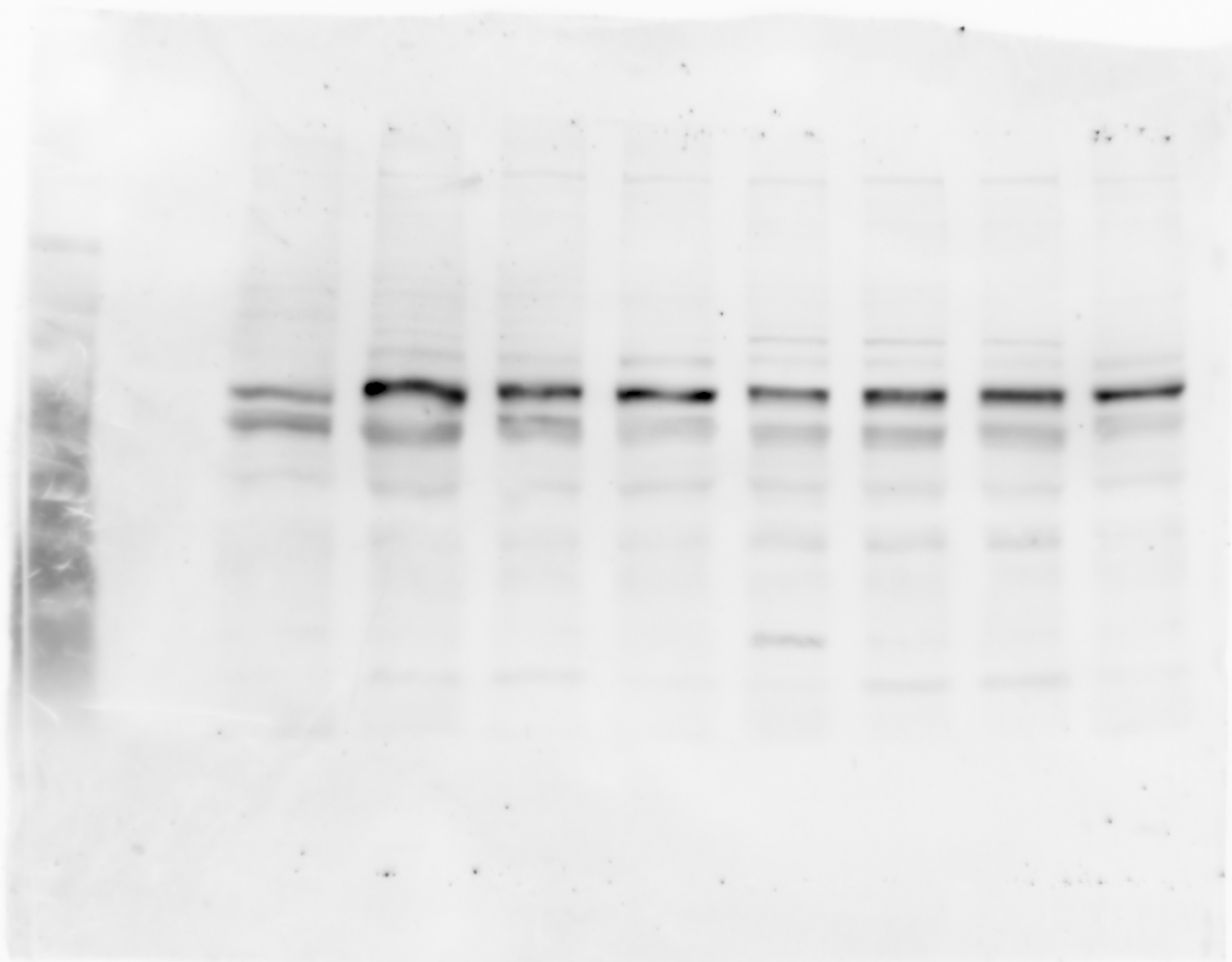

Supplement: Figure 6—source data 1. [file elife-75492-fig6-data1.zip › Figure 6B Source data 2.pdf]
